# Role of Systemic Treatment for Advanced/Metastatic Gastric Carcinoma in the Third-Line Setting: A Bayesian Network Analysis

**DOI:** 10.3389/fonc.2020.00513

**Published:** 2020-04-23

**Authors:** Wen-Tao Pan, Su-Na Zhou, Meng-Xian Pan, Qiu-Yun Luo, Lin Zhang, Da-Jun Yang, Miaozhen Qiu

**Affiliations:** ^1^State Key Laboratory of Oncology in South China, Department of Experimental Research, Collaborative Innovation Center for Cancer Medicine, Sun Yat-sen University Cancer Center, Guangzhou, China; ^2^State Key Laboratory of Oncology in South China, Department of Clinical Laboratory, Collaborative Innovation Center for Cancer Medicine, Sun Yat-sen University Cancer Center, Guangzhou, China; ^3^State Key Laboratory of Oncology in South China, Department of Medical Oncology, Collaborative Innovation Center for Cancer Medicine, Sun Yat-sen University Cancer Center, Guangzhou, China

**Keywords:** gastric cancer, systematic therapy, Bayesian network analysis, overall survival, progression free survival, safety

## Abstract

**Background:** Increasing evidences from phase II or III trials have proved that salvage systematic therapy, including chemotherapy, target therapy, or checkpoint inhibitor therapy can prolong survival in patients who do not succeed with second line therapy, yet there are no guidelines for the optimum third-line treatments. To compare the effectiveness and safety of current third-line therapies for metastatic Gastric Cancer (mGC), we conducted this network analysis.

**Methods:** Literature up to Sep 30, 2019 were systematically searched and analyzed by a Bayesian fixed-effect model.

**Results:** This study included seven randomized clinical trails which involved 2,655 patients. It turns out that for overall survival, nivolumab has the highest probability to be the optimal choice for overall survival (OS). For patients with no peritoneal metastases, the network meta-analysis showed that Nivolumab (HR:0.64; 95% CI: 0.48–0.85) and Trifluridine/tipiacil (HR:0.66; 95% CI: 0.51–0.86) were associated with significantly higher improvement in OS than placebo. However, patients with peritoneal metastases could not benefit from nivolumab, ramucirumab, or Trifluridine/tipiacil, when compared with a placebo. For progression-free survival, apatinib (850 mg) was the most likely candidate, followed by ramucirumab. Statistically, Apatinib (850 mg), Trifluridine/tipiacil, and SLC had higher incidences of high-grade adverse events (AEs) than placebo.

**Conclusion:** Our findings demonstrate that nivolumab has the best balance between acceptability and effectiveness in the third line therapy for mGC.

## Introduction

Gastric cancer (GC) has a high incidence rate all over the world, especially in eastern Asia. About 40% of GC patients were diagnosed with an advanced stage in China (1, 2). Several guidelines have recommended platinum plus fluropyimidine as first-line therapy for those patients who were advanced or had metastatic gastric cancer (3). For human epidermal growth factor receptor 2 (Her-2) positive advanced gastric cancer, adding trastuzumab to platinum-based chemotherapy has been considered as standard first line therapy (4). Both irinotecan and taxane have been accepted as second line therapy (5). Increasing evidence from phase II or III trials have proved that salvage systematic therapy, including chemotherapy, target therapy, or checkpoint inhibitor therapy can prolong survival in patients who do not succeed with second line therapy (6–10), when compared with a placebo. But it is hard for clinicians to determine the best therapy, because there are no head-to-head randomized clinical trials (RCTs) to evaluate the efficacy and safety among the available third line therapies. Besides, JAVELIN Gastric 300 study failed to demonstrate avelumab in third line setting was superior in OS improvement, compared with the physician's choice of chemotherapy (11). What if it were compared with a placebo? Will it be a possible treatment because of its similar mechanism to Nivolumab? In the present study, to answer these questions, we will use Bayesian network analysis to compare and summarize the clinical results and adverse events (AEs) of available third-line regimens for metastatic GC (mGC).

## Methods

### Search Strategy

Our research was conducted under the guidance of PRISMA extension statement for network meta-analysis. We performed a valid literature search on Cochrane Library, PubMed,Web of Science, and ClinicalTrials.gov for RCTs comparing at least two agents in third-line treatment of mGC in Sep, 2019. All literature was screened to ensure eligibility. Search terms such as chemotherapy, nivolumab, avelumab, apatinib etc., and search strategies are shown in Appendix 1 in Supplementary Information.

### Selection Criteria and Data Extraction

Only randomized controlled trials were included. Target mGC patients must have received at least two anti-tumor systemic therapies. Interventions included, but were not limited to: chemotherapy, nivolumab, avelumab, apatinib, ramucirumab, and Trifluridine/tipiracil (TAS102). Articles were excluded if the patient received first-line or second-line systemic therapy.

First, the main exclusion criteria for title and abstract screen of the studies obtained through systematic search were as follows: (1) non-RCT designs including preclinical experiments, case-control studies, and case reports (2) duplicates; (3) trials without comparisons; (4) commentaries and editorials; and (5) pooled analyses. After identification by title and abstract, the remaining studies were then reviewed in full text, and the exclusion criteria for the full text were as follows: (1) preliminary or repeated reports, (2) non-randomized clinical trials, (3) second-line treatment clinical trials. After the full-text review, we included 7 eligible RCTs and performed further analysis.

The primary outcome of the study was to measure overall survival (OS). Progression free survival (PFS) and high-grade (grade ≥ 3) drug related adverse events (AEs) (National Cancer Institute Common Toxicity Criteria version 3.0) were assessed as secondary outcomes.

The two reviewers (Wentao Pan and Miaozhen Qiu) searched and screened for studies with potential research at the level of title and abstract levels. Full texts were reviewed when abstracts were insufficient to assess trial eligibility. Then data on patient characteristics, treatment strategies, outcome definitions, and so forth were extracted into a standardized form by one reviewer (Wentao Pan), and validated by the other (Miaozhen Qiu). Disagreements arose and issues was resolved in consultation with a third reviewer (Suna Zhou). For example, should we include a single arm phase II study? After careful discussion, we decided not to include these studies. The Cochrane Handbook's Risk of Bias Assessment Tool for randomized trials was used to evaluate the quality of studies included.

### Data Synthesis and Statistical Analysis

In our analysis, we first tried Bayesian methods for random-effect model and fixed-effect model, but due to the absence of heterogeneities, only fixed-effect model could be run. Published hazard ratios (HRs) with 95% confidence intervals (CIs) were used as relevant parameters for OS and PFS assessment. Odds ratios (ORs) analyzed using available data attained from the trials were used to assess drug-related AEs. The results were assessed using contrast-based approach with 50,000 iterations in the training phase, for a total of three chains, for a total of 100,000. Operation code was offered in Appendix 2 in Supplementary Material. Based on the analysis of OS, PFS, and high-grade AEs, the distribution of ranking probabilities to rank treatments was used and exported directly from OpenBUGS version 3.2.2.

Apart from the calculation of OR using Stata v.13 (StataCorp, College Station, TX, USA), most of the data analyses were performed using OpenBUGS version 3.2.2. Under Stata v.13, the heterogeneity between studies was assessed by the Q-test and I2 statistic, and heterogeneity was considered when *P* < 0.10 and *I*^2^ > 50%.

## Results

### Literature Search

**Figure 1:**
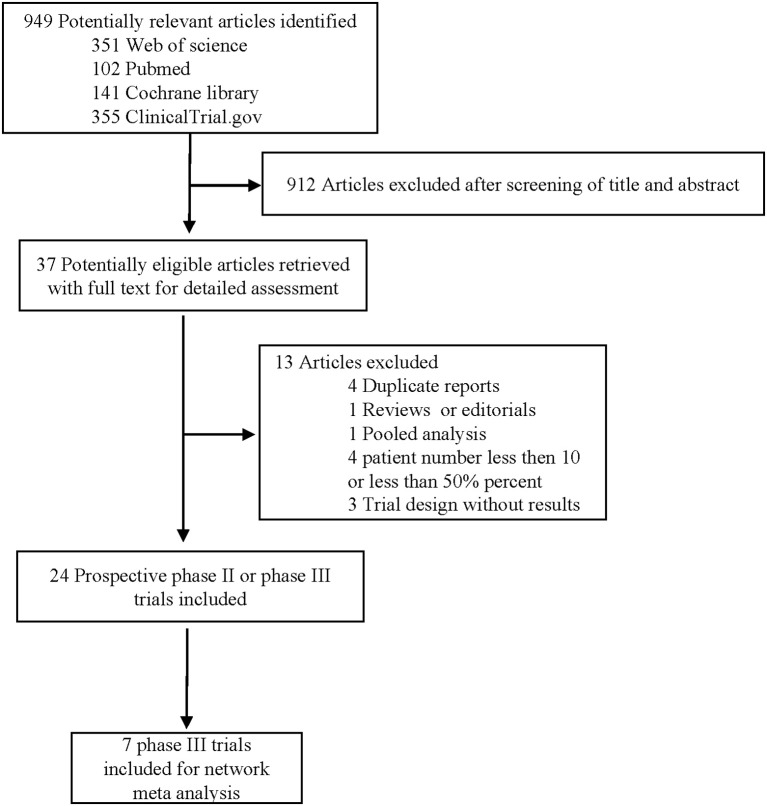
Literature search and selection.

Through our literature search, we reviewed 949 potential studies, of which 912 were excluded after screening titles and abstracts (Figure 1). Through a thoughtful review of the remaining 24 studies, 7 phase III RCTs (2,655 patients) were included in this network meta-analysis (Table 1), with an average of 189 (range 69–337) per group and at least 100 cases per group.

**Table 1:** Studies included in the meta-analysis.

**Study**	**Number of patients**	**Age (years) median(range)**	**Sex (% male)**	**Median OS in months (range)**	**OS HR (95%CI)**	**Median PFS In months (range)**	**PFS HR (95% CI)**	**HighGrade AE, %**	**Phase of the trial**
Li et al. (12)									3
Apatinib (800 mg)	176	58 (23–71)	75	6.5 (4.8–7.6)	0.7 (0.5–0.9)	2.6 (2–2.9)	0.4 (0.3–0.6)	69.2	
Placebo	91	58 (28–70)	75.8	4.7 (3.6–5.4)	1 (Ref)	1.8 (1.4–1.9)	1 (Ref)	42.9	
Kang et al. (8)									3
Nivolumab	330	62 (54–69)	69	5.3 (4.6–6.4)	0.6 (0.5–0.8)	1.6 (1.5–2.3)	0.6 (0.5–0.8)	10	
Placebo	163	61 (53–68)	73	4.1 (3.4–4.9)	1 (Ref)	1.5 (1.4–1.5)	1 (Ref)	4	
Fuchs et al. (13)									3
Ramucirumab	238	60 (52–67)	71	5.2 (2.3–9.9)	0.8 (0.6–0.9)	2.1 (1.3–4.2)	0.5 (0.4–0.6)	57	
Placebo	117	60 (51–71)	68	3.8 (1.7–7.1)	1 (Ref)	1.3 (1.1–2.1)	1 (Ref)	58	
Shitara et al. (14)									3
Trifluridine/tipiracil	337	64 (56–70)	75	5.7 (4.8–6.2)	0.7 (0.6–0.9)	2 (1.9–2.3)	0.6 (0.5–0.7)	80	
Placebo	170	63 (56–69)	69	3.6 (3.1–4.1)	1 (Ref)	1.8 (1.7–1.9)	1 (Ref)	58	
Ryu et al. (15)									3
Apatinib(700 mg)	308	60 (21–91)	78.3	5.8	0.9 (0.7–1.2)	2.8	0.6 (0.5–0.8)	47.6	
Placebo	105	61 (27–82)	73.7	5.1	1 (Ref)	1.8	1 (Ref)	43.7	
Kang et al. (9)									3
SLC	133	56 (31–83)	70	5.3 (4.1–6.5)	0.7 (0.5–0.9)	NR	NR	87	
Placebo	69	56 (32–74)	64	3.8 (3.1–4.5)	1 (Ref)			75	
Bang et al. (11)									3
avelumab	185	59 (29–86)	75.7	4.6 (3.6–5.7)	1.1 (0.9–1.4)	1.4 (1.4–1.5)	1.7 (1.4–2.2)	9.7	
SLC	186	61 (18–82)	68.3	5 (4.5–6.3)	1 (Ref)	2.7 (1.8–2.8)	1 (Ref)	38.9	

### Charateristics of Included Studies and Network Assumptions

**Figure 2:**
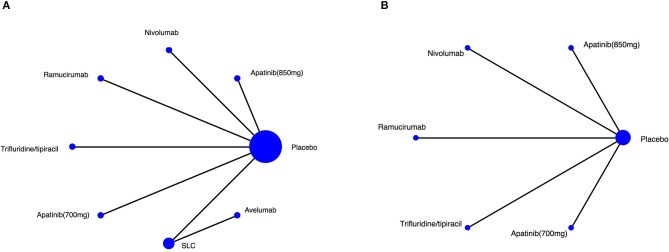
Network of the comparisons for the Bayesian network meta-analysis. Network plot for **(A)** OS, **(B)** PFS. The size of every treatment node corresponds to the patient's number of randomly assigned treatment. SLC, salvage chemotherapy.

**Figure 3:**
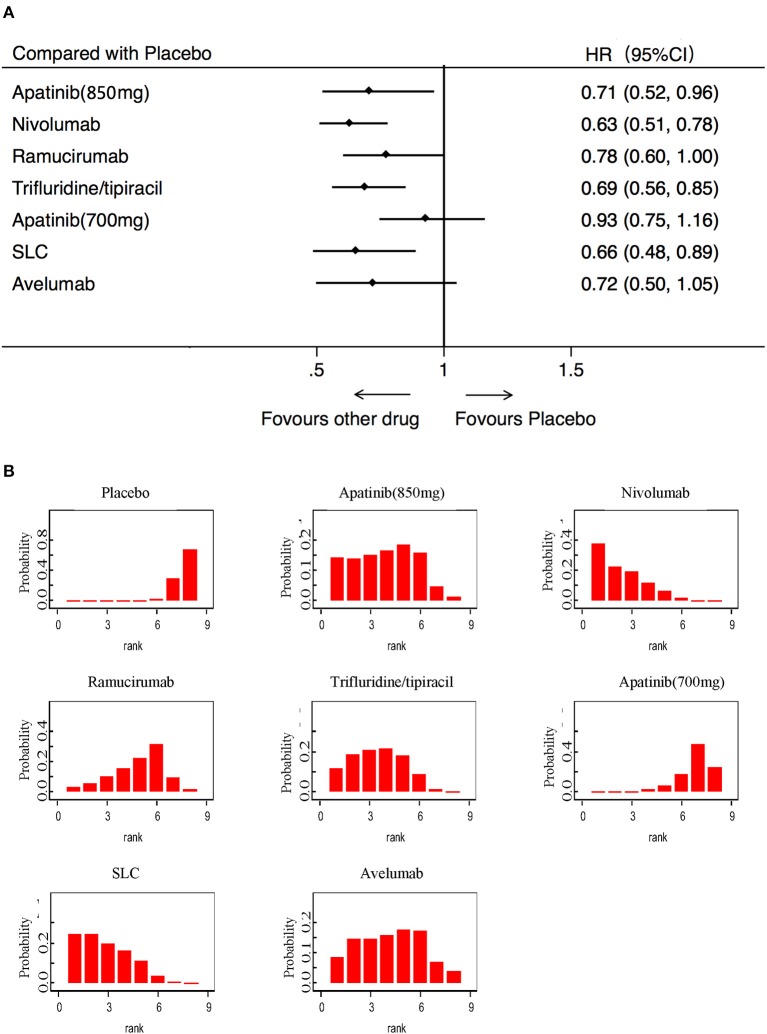
Pooled hazard ratios for overall survival. **(A)** Forest plot, with placebo as the comparator; A fixed effect model was adopted due to non-significant heterogeneity of publications (*I*^2^ = 7.1%, *p* = 0.374). **(B)** Ranking of treatments in terms of overall survival. Rankograms were drawn according to distribution of the ranking probabilities. HR, hazard ratio; CI, credible interval; Numbers in parentheses indicate 95% credible intervals. SLC, salvage chemotherapy.

The RCT features were shown in Table 1, and no significant differences were observed in study characteristics, with a median age of 60 years and 72.4% of male patients (Supplementery Figure 1). The comparison network was represented as a network diagram with eight and six treatments for those with OS data (Figure 2A) and those with PFS data (Figure 2B), respectively. We firstly tried a Bayesian approach with random-effect model and fixed-effect model, but there was no heterogeneities due to the included studies. Therefore, only analysis with fixed-effect model could be attained, and in each analysis, the three chains showed good fusion (Supplementery Figures 2, 3).

### Overall Survival (OS)

**Figure 4:**
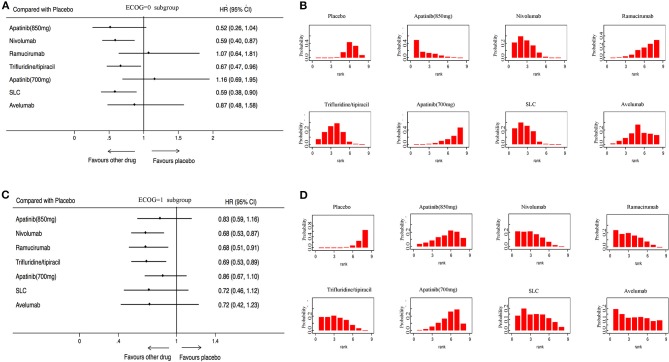
Pooled hazard ratios for overall survival in subgroup patients. **(A)** Forest plot, with placebo as the comparator in patients with ECOG = 0; A fixed effect model was adopted due to non-significant heterogeneity of publications (*I*^2^ = 1.0%, *p* = 0.417). **(B)** Ranking of treatments in terms of overall survival in patients with ECOG = 0. **(C)** Forest plot, with placebo as the comparator in patients with ECOG = 1; A fixed effect model was adopted due to non-significant heterogeneity of publications (*I*^2^ = 0.0%, *p* = 0.854). **(D)** Ranking of treatments in terms of overall survival in patients with ECOG = 1. Rankograms were drawn according to distribution of the ranking probabilities. HR, hazard ratio; CI, credible interval. Numbers in parentheses indicate 95% credible intervals. SLC, salvage chemotherapy.

OS was analyzed in 7 studies (2,655 patients) with 8 third-line systemic therapies. Network meta-analysis indicated that Apatinib (850 mg), Nivolumab, Trifluridine/tipiacil, and SLC were significantly associated with improved OS compared with a placebo (Figure 3A). According to the ranking results, Nivolumab was most likely to be the best choice for OS, and a placebo was considered the least likely (Figure 3B). In patients with ECOG = 0, Nivolumab, Trifluridine/tipiacil, and SLC improved significantly more than the placebo with OS (Figure 4A), juxtaposed first with Apatinib, Nivolumab, and SLC (Figure 4B). In patients with ECOG = 1, Nivolumab, Ramucirumab, and Trifluridine/tipiacil improved OS significantly compared with placebo (Figure 4C), with Nivolumab, Ramucirumab, and Trifluridine/tipiacil juxtaposed first (Figure 4D).

Further, we analyze patients with or without more than 2 metastatic sites in 6 studies for OS. The network meta-analysis showed that Apatinib (850 mg) (HR:0.70; 95% CI:0.50–0.99), Trifluridine/tipiacil (HR:0.68; 95% CI: 0.49–0.95), and SLC (HR:0.55; 95% CI: 0.33–0.93) were associated with significantly higher improvement in OS than a placebo (Supplementery Figure 4A), with SLC ranking the first (Supplementery Figure 4B). For those with more than 2 metastatic sites, we found that Nivolumab (HR:0.62; 95% CI:0.49–0.79), Trifluridine/tipiacil (HR:0.71; 95% CI: 0.54–0.94), and SLC(HR:0.63; 95% CI: 0.42–0.94) were associated with significantly higher improvements in OS than a placebo (Supplementery Figure 4C), with Nivolumab and SLC ranking the highest (Supplementery Figure 4D).

**Figure 5:**
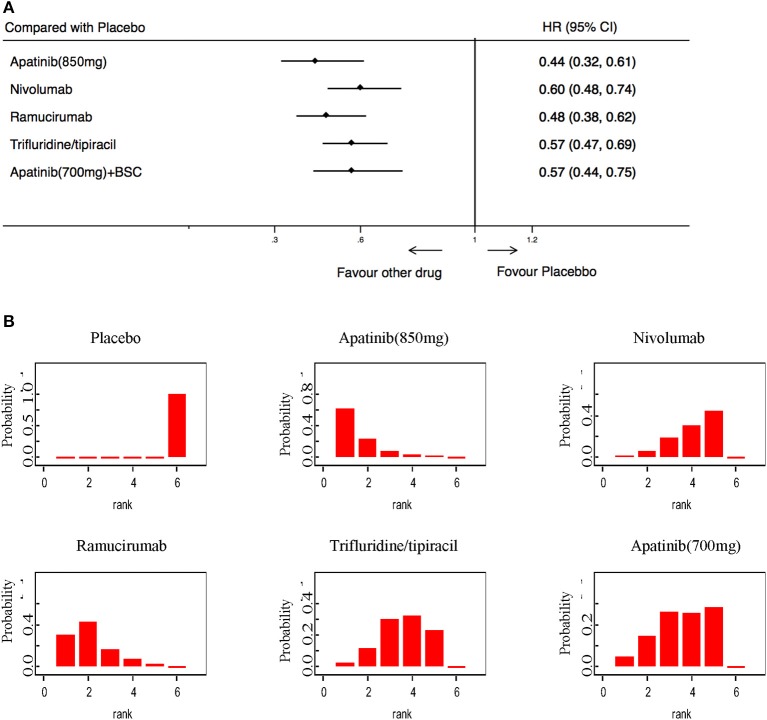
Pooled hazard ratios for progression-free survival. **(A)** Forest plot, with placebo as the comparator; A fixed effect model was adopted due to non-significant heterogeneity of publications (*I*^2^ = 0.0%, *p* = 0.437). **(B)** Ranking of treatments in terms of progression-free survival. Rankograms were drawn according to distribution of the ranking probabilities. HR, hazard ratio; CI, credible interval. Numbers in parentheses indicate 95% credible intervals.

Meanwhile, 309 patients with no measurable disease were used in 5 studies for OS. The network meta-analysis showed that Trifluridine/tipiacil (HR:0.21; 95% CI: 0.09–0.50) and SLC(HR:0.36; 95% CI: 0.20–0.67) were associated with significantly higher improvements in OS than a placebo (Supplementery Figure 5A), with Trifluridine/tipiacil ranking the highest (Supplementery Figure 5B). For those with a measurable disease, we found that Nivolumab (HR:0.64; 95% CI:0.49–0.83) and TAS102 (HR:0.74; 95% CI: 0.59–0.93) were associated with significantly higher improvements in OS than a placebo (Supplementery Figure 5C), with Nivolumab ranking the highest (Supplementery Figure 5D).

**Figure 6:**
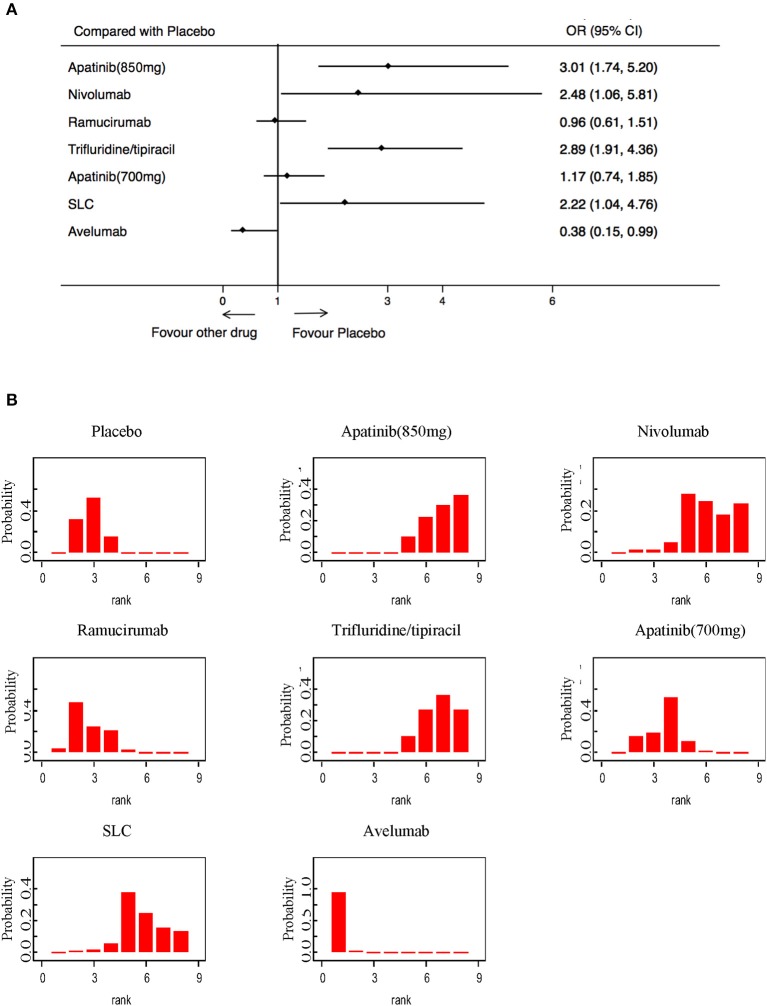
Pooled hazard ratios for high-grade adverse events. **(A)** Forest plot, with placebo as the comparator; significant heterogeneity of publications was seen (*I*^2^ = 77.1%, *p* = 0.000). **(B)** Ranking of treatments in terms of high-grade adverse events. Rankograms were drawn according to distribution of the ranking probabilities. HR, hazard ratio; CI, credible interval. Numbers in parentheses indicate 95% credible intervals.

Lastly, we found that patients with or without peritoneal metastases have different responses to treatment. Three studies with corresponding data were analyzed. For patients with no peritoneal metastases, the network meta-analysis showed that Nivolumab (HR:0.64; 95% CI: 0.48–0.85) and Trifluridine/tipiacil (HR:0.66; 95% CI: 0.51–0.86) were associated with significantly higher improvements in OS than a placebo (Supplementery Figure 6A), with Nivolumab ranking the highest (Supplementery Figure 6B). Patients with peritoneal metastases could not benefit from Nivolumab, Ramucirumab, or Trifluridine/tipiacil, when compared with a placebo (Supplementery Figures 6C,D).

### Secondary Endpoints

In terms of PFS, 5 trials including 2,035 patients were available for evaluation. The results showed that Apatinib (850 mg), Nivolumab, Ramucirumab, Trifluridine/tipiacil, and Apatinib (700 mg) were statistically superior to a placebo (Figure 5A). PFS rankings showed that Apatinib (850 mg) was the most likely to be preferred. In second place was ramucirumab, followed closely by Nivolumab, Trifluridine/tipiacil, and Apatinib (700 mg) (Figure 5B).

High-grade treatment-related toxicities were analyzed in 2,608 patients in 7 RCTs. Compared with a placebo, only the Avelumab (OR: 0.38; 95% CI: 015–099) was associated with a lower incidence of high-grade AEs (Figure 6A). Apatinib (850 mg), Trifluridine/tipiacil, and SLC showed statistically higher rates of high-grade AEs than a placebo (Figure 6A). Avelumab was the most tolerable of all treatments, while Apatinib (850 mg) was the most toxic (Figure 6B).

### Publication Bias and Risk of Bias

To test the robustness of the significant results, we performed a network heterogeneity test, only to find homogeneity. By comparing the adjusted funnel plots of OS and PFS, we reported a symmetric distribution (Supplementery Figure 7), indicating the absence of small study effects and publication bias. Of the included studies, the methodological quality was good, with only one trial verbally reported and not accurately assessing its risk bias. Overall, all remaining studies had no significant high risk of bias with respect to random sequence generation, allocation concealment, incomplete outcome data, and selective reporting of outcomes (Supplementery Figure 8).

## Discussion

The network meta-analysis was based on 7 trials involving 2,655 patients, and 7 placebo-controlled mGC third-line treatments. There are several major findings. First of all, nivolumab may be the optimal choice for OS improvement. Secondly, in terms of PFS, Apatinib (850 mg) may be the first choice followed by ramucirumab, Nivolumab, Trifluridine/tipiacil, and Apatinib (700 mg). Finally, Avelumab had the best safety profile followed by ramucirumab, Apatinib (700 mg), Nivolumab, SLC, Trifluridine/tipiacil, and Apatinib (850 mg).

A systematic review and meta-analysis of third-line chemotherapy in advanced GC concluded that patients with advanced gastric cancer who underwent third line chemotherapy had superior results to those who received optimal supportive care (10), but there is no comparison among different third line treatment, such as apatinib, nivolumab, and trifluridine/tipiacil. Based on the present analysis, nivolumab appeared to be the best option for OS. Except for nivolumab, another immune checkpoint inhibitor, pembrolizumab, was approved for patients with recurrent locally advanced or metastatic PD-L1 positive GC. The results were based on KEYNOTE-059 study (7). Since KEYNOTE-059 was an open label, phase 2 trial, it was not included in our meta-analysis. As we know, Avelumab is an anti-PD-L1 antibody, blocking the combination of PD-1 and PD-L1, and sharing a similar MOA with Nivolumab. Compared with chemotherapy, it failed to improve OS. In our study, although compared with a placebo, it still could not get OS benefit. These indicated that anti-PD-L1 antibody and anti-PD-1 antibody played different roles in anti-tumor.

Peritoneal metastasis is related to poor prognosis in mGC patients (16). PHOENIX-GC Trial showed that intraperitoneal paclitaxel plus systemic chemotherapy did not improve the prognosis of GC patients with peritoneal metastasis (17). In the subgroup analysis of the present study, we found that patients with peritoneal metastases could not benefit from available third line therapy including Nivolumab, Ramucirumab, or Trifluridine/tipiacil, when compared with a placebo. Though it is disappointing, our results remind us that best supportive care is enough for GC patients with peritoneal metastases when they do not respond to second line therapy.

In terms of PFS, apatinib (850 mg), ramucirumab, Nivolumab, Trifluridine/tipiacil, and apatinib (700 mg) were statistically superior to a placebo. Though we observed the OS benefit for Nivolumab over apatinib, the different effectiveness of these treatments relative to OS and PFS were worth noting. Though immune checkpoint inhibitors can bring survival benefit, the response rate and PFS of immune checkpoint inhibitors are not high. This phenomenon has also been observed in other phase 3 randomized clinical trial in gastric cancer (18) or in esophageal carcinoma (8, 19). Though apatinib (850 mg) prolongs PFS and OS, it has the poorest safety profile among the available third line therapies. When the dose of apatinib decreased to be 700 mg, it is well-tolerated, but the OS was not significantly different with a placebo.

Our research advantages are as follows. Firstly, this research is by far the most comprehensive network analysis for evaluating the effectiveness and safety of currently available third-line systemic therapies for mGC. In addition, we could integrate the available information from RCTs, indirectly evaluate multiple treatments without head-to-head trials, and provide a hierarchical order for treatments based on OS, PFS, and high-grade AEs in mGC by using Bayesian network meta-analysis. Although theoretically it may have resulted in the expansion of type I error in the Bayesian network meta-analysis, the type I error rate proved to be controllable (20). Finally, the evaluation of effectiveness and safety provides a new perspective for different systemic therapies, and probably provides guidance for patients and clinicians to make treatment decisions and design future comparative trials. Nevertheless, we should also consider the limitations of our research. The main limitation of our study was research design, because 6 out of 7 studies took placebo as reference treatment. Only fixed-effect model could be used, excluding random-effect model, meta-regression analysis, and sensitivity analysis. Fortunately, these 7 high-quality phase III studies included were not heterogeneous in publication, and there were no significant differences in research features. In addition, there were no confounding factors such as prognostic risk categories and peritoneal metastasis that might affect the benefits of systemic therapy.

## Conclusions

Our results indicated that nivolumab could provide the best OS benefit for mGC. Apatinib (850 mg) is the best choice for PFS. Nivolumab might also be a potential option for mGC, as it had the most favorable balance between effectiveness and safety. Given the limitations of this study, more head-to-head comparative RCTs are needed to verify our conclusions.

## Author Contributions

MQ, D-JY, and LZ: conception and design of this study. W-TP, S-NZ, and MQ: data extraction. M-XP and Q-YL: data analysis and statistical analysis. W-TP and MQ: manuscript writing. All authors: reviewed and approved.

## Conflict of Interest

The authors declare that the research was conducted in the absence of any commercial or financial relationships that could be construed as a potential conflict of interest.
