# Supplementary material for: Role of Systemic Treatment for Advanced/Metastatic Gastric Carcinoma in the Third-Line Setting: A Bayesian Network Analysis
Source: Front Oncol. 2020 Apr 23;10:513. doi: 10.3389/fonc.2020.00513 (PMC7191061; doi:10.3389/fonc.2020.00513)

Appendix 1. Search strategy.

**Pubmed:**

((((((((((((((((((((((((((((((((((((((((((((((((((((((((((((SHR-1210[Title/Abstract]) OR camrelizumab[Title/Abstract]) OR BGB-A317[Title/Abstract]) OR Tislelizumab[Title/Abstract]) OR toripalimab[Title/Abstract]) OR JS001[Title/Abstract]) OR sintilimab[Title/Abstract]) OR IBI308[Title/Abstract]) OR nivolumab[Title/Abstract]) OR ipilimumab[Title/Abstract]) OR pembrolizumab[Title/Abstract]) OR avelumab[Title/Abstract]) OR atezolizumab[Title/Abstract]) OR PD-1 antibody[Title/Abstract]) OR PD-L1 antibody[Title/Abstract]) OR immune checkpoint blockade[Title/Abstract]) OR immunotherapy[Title/Abstract]) OR Apatinib[Title/Abstract]) OR Irinotecan[Title/Abstract]) OR Paclitaxel[Title/Abstract]) OR taxane[Title/Abstract]) OR ramucirumab[Title/Abstract]) OR regorafenib[Title/Abstract]) OR TAS102[Title/Abstract]) OR Trifluridine[Title/Abstract]) OR tipiracil[Title/Abstract]) OR docetaxol[Title/Abstract])) OR Simvastatin[Title/Abstract]) OR S-1[Title/Abstract]) OR 5-FU[Title/Abstract]) OR cisplatin[Title/Abstract]) OR TAS-102[Title/Abstract]) OR Reconvan[Title/Abstract]) OR Oxaliplatin[Title/Abstract]) OR Andecaliximab[Title/Abstract]) OR Paclitaxel[Title/Abstract]) OR epirubicin[Title/Abstract]) OR Everolimus[Title/Abstract]) OR Krestin[Title/Abstract]) OR Capecitabine[Title/Abstract]) OR Bevacizumab[Title/Abstract]) OR DWJ1319[Title/Abstract]) OR FOLFIRI[Title/Abstract]) OR MTX[Title/Abstract]) OR Irinotecan[Title/Abstract]) OR ECC[Title/Abstract]) OR mitomycin[Title/Abstract]) OR doxifluridine[Title/Abstract]) OR XRP6976[Title/Abstract]) OR Cetuximab[Title/Abstract]) OR G17DT[Title/Abstract]) OR Lapatinib[Title/Abstract]) OR Onartuzumab[Title/Abstract]) OR epoetin alfa[Title/Abstract]) OR Enoxaparin[Title/Abstract]) OR epirubicin[Title/Abstract]) OR Andecaliximab[Title/Abstract])) AND third line[Title/Abstract]) AND ((•Neoplasm, Stomach •Stomach Neoplasm •Neoplasms, Stomach •Gastric Neoplasms •Gastric Neoplasm •Neoplasm, Gastric •Neoplasms, Gastric •Cancer of Stomach •Stomach Cancers •Gastric Cancer •Cancer, Gastric •Cancers, Gastric •Gastric Cancers •Stomach Cancer •Cancer, Stomach •Cancers, Stomach •Cancer of the Stomach •Gastric Cancer, Familial Diffuse) AND "Stomach Neoplasms"[Mesh])

**Cochrane Library:**

"SHR-1210 OR camrelizumab OR BGB-A317 OR Tislelizumab OR toripalimab OR JS001 OR sintilimab OR IBI308 OR nivolumab OR ipilimumab OR pembrolizumab OR avelumab OR atezolizumab OR PD-1 antibody OR PD-L1 antibody OR immune checkpoint blockade OR immunotherapy OR Apatinib OR Irinotecan OR Paclitaxel OR taxane OR ramucirumab OR regorafenib OR TAS102 OR Trifluridine OR tipiracil OR docetaxol ) OR Simvastatin OR S-1 OR 5-FU OR cisplatin OR TAS-102 OR Reconvan OR Oxaliplatin OR Andecaliximab OR Paclitaxel OR epirubicin OR Everolimus OR Krestin OR Capecitabine OR Bevacizumab OR DWJ1319 OR FOLFIRI OR MTX OR Irinotecan OR ECC OR mitomycin OR doxifluridine OR XRP6976 OR Cetuximab OR G17DT OR Lapatinib OR Onartuzumab OR epoetin alfa OR Enoxaparin OR epirubicin OR Andecaliximab" anywhere and "third line" anywhere and "Neoplasm Stomach OR Stomach Neoplasm OR Neoplasms Stomach OR Gastric Neoplasms OR Gastric Neoplasm OR Neoplasm OR Gastric Neoplasms OR Gastric Cancer of Stomach OR Stomach Cancers OR Gastric Cancer OR Cancer OR Gastric Cancers OR Gastric Gastric OR Cancers OR Stomach Cancer OR Stomach Cancers OR Stomach Cancer of the Stomach Gastric Cancer Familial Diffuse OR Stomach Neoplasms

Web of science:

TS=(SHR-1210 OR camrelizumab OR BGB-A317 OR Tislelizumab OR toripalimab OR JS001 OR sintilimab OR IBI308 OR nivolumab OR ipilimumab OR pembrolizumab OR avelumab OR atezolizumab OR PD-1 antibody OR PD-L1 antibody OR immune checkpoint blockade OR immunotherapy OR Apatinib OR Irinotecan OR Paclitaxel OR taxane OR ramucirumab OR regorafenib OR TAS102 OR Trifluridine OR tipiracil OR docetaxol OR Simvastatin OR S-1 OR 5-FU OR CISPLATIN OR TAS-102 OR Reconvan OR Oxaliplatin OR Andecaliximab OR Paclitaxel OR epirubicin OR Everolimus OR Krestin OR Capecitabine OR Bevacizumab OR DWJ1319 OR FOLFIRI OR MTX OR Irinotecan OR ECC OR mitomycin OR doxifluridine OR XRP6976 OR Cetuximab OR G17DT OR Lapatinib OR Onartuzumab OR epoetin alfa OR Enoxaparin OR epirubicin OR Andecaliximab)

TS=(third line)

TS=(Stomach Neoplasm OR Neoplasms, Stomach OR Gastric Neoplasms OR Gastric Neoplasm OR Neoplasm, Gastric OR Neoplasms, Gastric OR Cancer of Stomach OR Stomach Cancers OR Gastric Cancer OR Cancer, Gastric OR Cancers, Gastric OR Gastric Cancers OR Stomach Cancer OR Cancer, Stomach OR Cancers, Stomach OR Cancer of the Stomach OR Gastric Cancer OR Stomach Neoplasms)

**ClinicalTrials.gov:**

Gastric carcinoma | Completed Studies | Interventional Studies | Gastric Cancer |phase 2, phase 3 (http://clinicaltrials.gov/)

**Appendix 2.** OpenBUGS code for fixed-effect model and random-effect model.

**Code for fixed-effect model:**

model

{

#Define Prior Distributions

#On tx effect mean

beta[1] < -0

for (tt in 2:nTx){

beta[tt]~dnorm(0,1.0E-6)

}

#On individual study baseline effect

for(ss in 1:nStudies){

alpha[ss] ~ dnorm(0,1.0E-6)

}

#Fit data

#For hazard ratio reporting studies

for(ii in 1:LnObs ){

Lmu[ii] < - alpha[Lstudy[ii]]*multi[ii] + beta[Ltx

[ii]] - beta[Lbase[ii]]

Lprec[ii] < - 1/pow(Lse[ii],2)

Lmean[ii] ~ dnorm(Lmu[ii],Lprec[ii])

}

# Calculate HRs

for (hh in 1:nTx) {

hr[hh] < -exp(beta[hh])

}

# Ranking plot

for (ll in 1:nTx) {

for (mm in 1:nTx) {

rk[ll,mm] < - equals(ranked(beta[],mm),beta[ll])

}

}

}

# Data

# Data set descriptors

list(LnObs =, nTx =, nStudies = )

# Log hazard ratio and log hazard data

Lstudy[] Ltx[] Lbase[] Lmean[] Lse[] multi[]

**Code for random-effect model:**

model

{

#Define Prior Distributions

#on random tx effect variance

sd~dunif(0,5)

reTau < - 2/pow(sd,2)

#On tx effect mean

beta[1] < -0

for (tt in 2:nTx){

beta[tt]~dnorm(0,1.0E-6)

}

#On individual study baseline effect

for(ss in 1:nStudies){

alpha[ss] ~ dnorm(0,1.0E-6)

}

#Define random effect

for (ss in 1:nStudies){

for(tt in 1:nTx){

re[ss,tt]~dnorm(0,reTau)

}

}

#Fit data

#For hazard ratio reporting studies

for(ii in 1:LnObs ){

Lmu[ii] < - alpha[Lstudy[ii]]*multi[ii] + re[Lstudy

[ii],Ltx[ii]] -

re[Lstudy[ii],Lbase[ii]] + beta[Ltx[ii]] - beta

[Lbase[ii]]

Lprec[ii] < - 1/pow(Lse[ii],2)

Lmean[ii] ~ dnorm(Lmu[ii],Lprec[ii])

}

# Calculate HRs

for (hh in 2:nTx) {

hr[hh] < -exp(beta[hh])

}

# Ranking plot

for (ll in 1:nTx) {

for (mm in 1:nTx) {

rk[ll,mm] < - equals(ranked(beta[],mm),beta[ll])

}

}

}

# Data

# Data set descriptors

list(LnObs =, nTx =, nStudies =)

# Log hazard ratio and log hazard data

Lstudy[] Ltx[] Lbase[] Lmean[] Lse[] multi[]

Fig. S1 (A)Box plots showing the distribution of the median age (years) of the patients across the studies included. (B)Box plots showing the distribution of the sex ratio (male %) of the patients across the 7 studies.


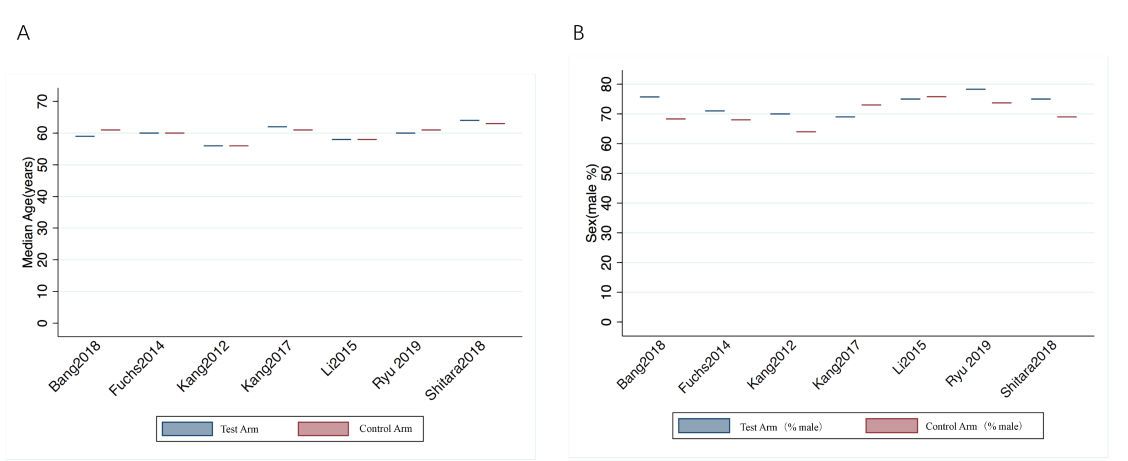


**Fig. S2** **Traces run by OpenBUGS (endpoint analysis).**


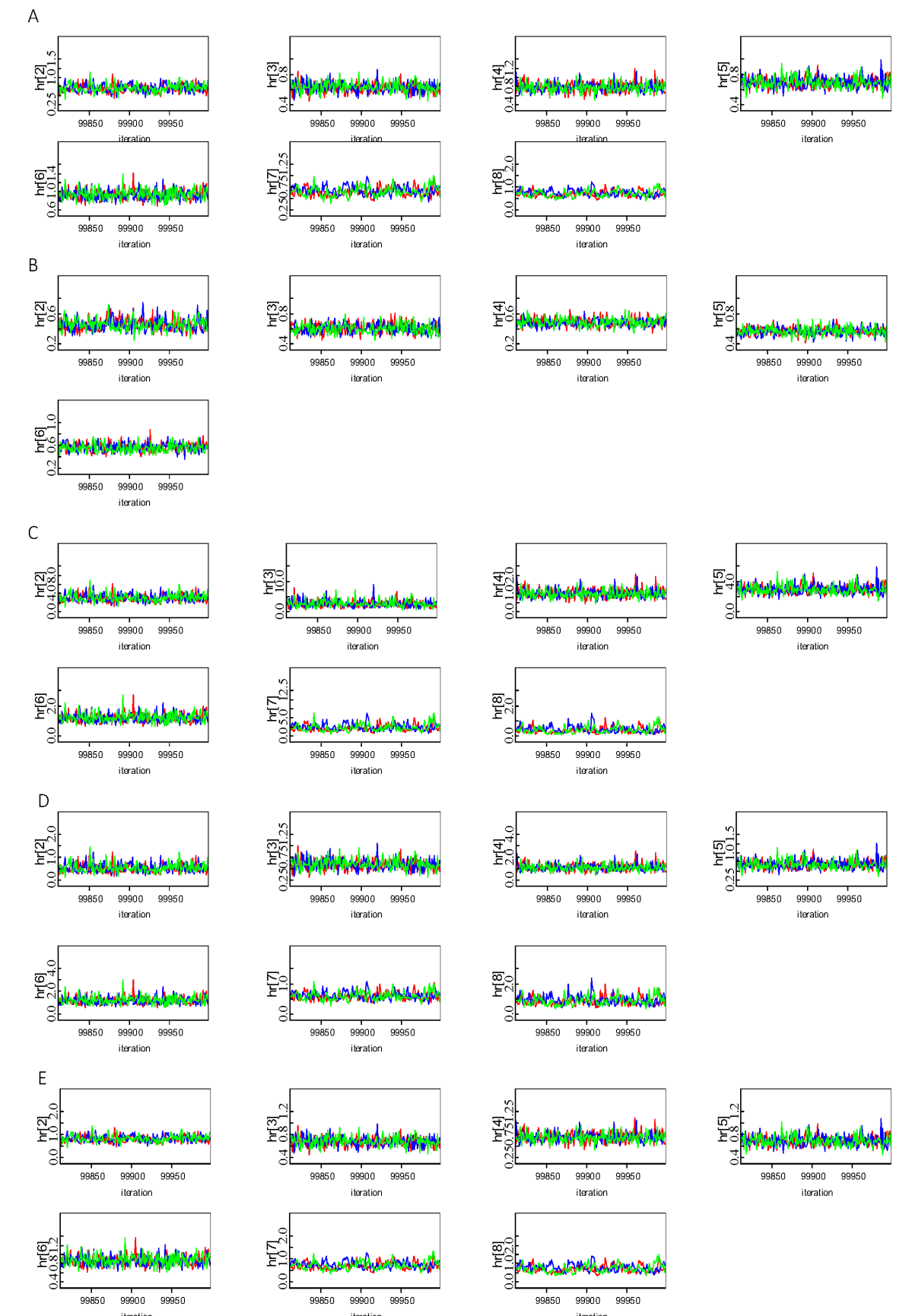


(A) Traces shown when hazard ratios (HRs) with 95% credible intervals (CIs) were used to assess OS. hr[2]= Apatinib(850mg) vs. Placebo, hr[3]= Nivolumab vs.Placebo, hr[4]= Ramucirumab vs. Placebo, hr[5]= Trifluridine/tipiracil vs. Placebo, hr[6]= Apatinib(700mg) vs. Placebo, hr[7]= SLC vs. Placebo, hr[8]= Avelumab vs. SLC; SLC=salvage chemotherapy.

(B) Traces shown when hazard ratios (HRs) with 95% credible intervals (CIs) were used to assess PFS. hr[2]= Apatinib(850mg) vs. Placebo, hr[3]= Nivolumab vs.Placebo, hr[4]= Ramucirumab vs. Placebo, hr[5]= Trifluridine/tipiracil vs. Placebo, hr[6]= Apatinib(700mg) vs. Placebo.

(C)Traces shown when Odds ratios (ORs) with 95% credible intervals (CIs) were used to assess high-grade adverse events. hr[2]= Apatinib(850mg) vs. Placebo, hr[3]= Nivolumab vs.Placebo, hr[4]= Ramucirumab vs. Placebo, hr[5]= Trifluridine/tipiracil vs. Placebo, hr[6]= Apatinib(700mg) vs. Placebo, hr[7]= SLC vs. Placebo, hr[8]= Avelumab vs. SLC; SLC=salvage chemotherapy.

(D)Traces shown when hazard ratios (HRs) with 95% credible intervals (CIs) were used to assess OS for patients with ECOG=0. hr[2]= Apatinib(850mg) vs. Placebo, hr[3]= Nivolumab vs.Placebo, hr[4]= Ramucirumab vs. Placebo, hr[5]= Trifluridine/tipiracil vs. Placebo, hr[6]= Apatinib(700mg) vs. Placebo, hr[7]= SLC vs. Placebo, hr[8]= Avelumab vs. SLC; SLC=salvage chemotherapy.

(E)Traces shown when hazard ratios (HRs) with 95% credible intervals (CIs) were used to assess OS for patients with ECOG=1. hr[2]= Apatinib(850mg) vs. Placebo, hr[3]= Nivolumab vs.Placebo, hr[4]= Ramucirumab vs. Placebo, hr[5]= Trifluridine/tipiracil vs. Placebo, hr[6]= Apatinib(700mg) vs. Placebo, hr[7]= SLC vs. Placebo, hr[8]= Avelumab vs. SLC; SLC=salvage chemotherapy.

**Fig. S3 Traces run by OpenBUGS (exploratory test analysis).**


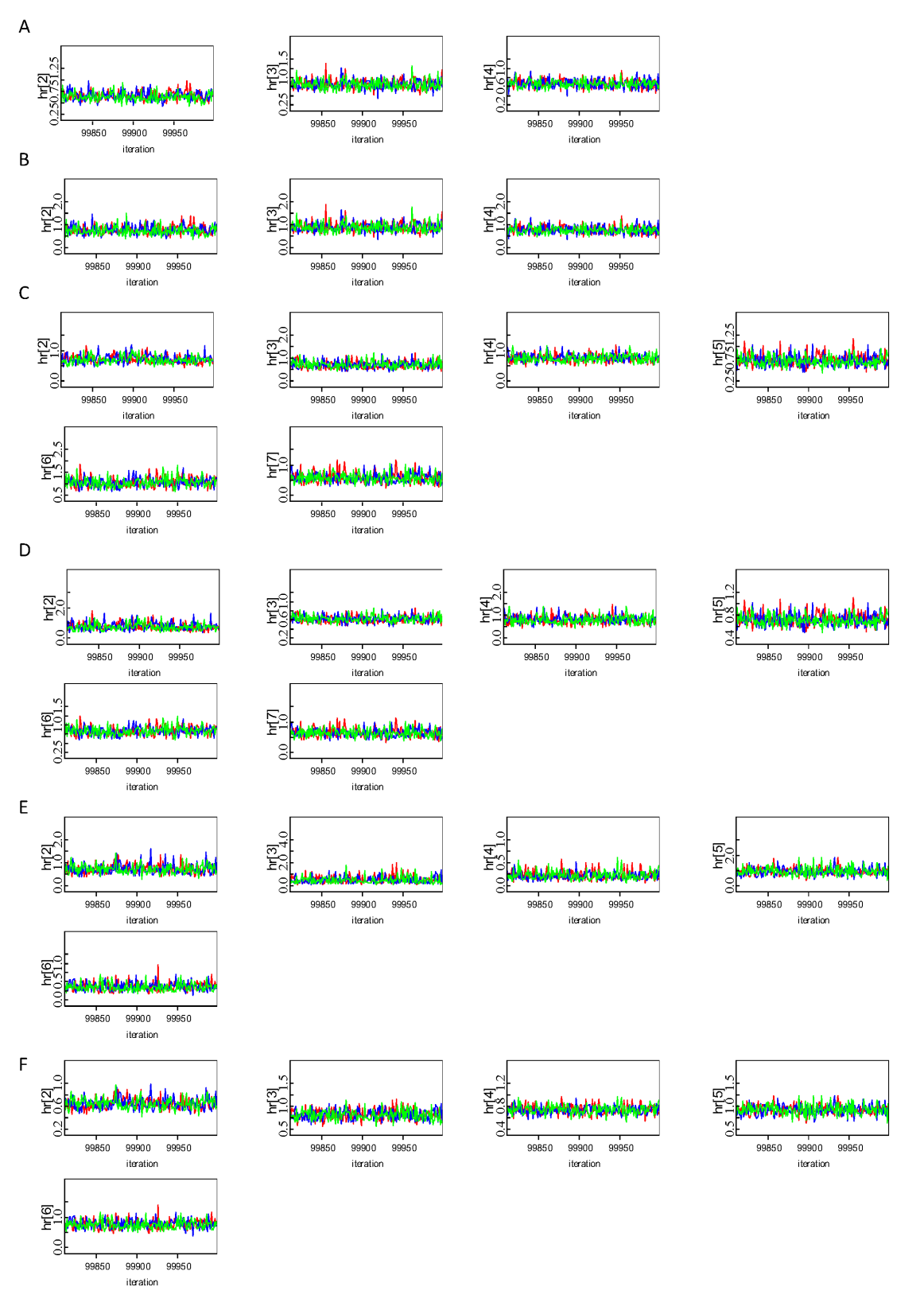


(A)Traces shown when hazard ratios (HRs) with 95% credible intervals (CIs) were used to assess OS for patients with no peritoneal metastasis. hr[2]= Nivolumab vs.Placebo, hr[3]= Ramucirumab vs. Placebo, hr[4]= Trifluridine/tipiracil vs. Placebo.

(B)Traces shown when hazard ratios (HRs) with 95% credible intervals (CIs) were used to assess OS for patients with peritoneal metastasis. hr[2]= Nivolumab vs.Placebo, hr[3]= Ramucirumab vs. Placebo, hr[4]= Trifluridine/tipiracil vs. Placebo.

(C)Traces shown when hazard ratios (HRs) with 95% credible intervals (CIs) were used to assess OS for patients with no more than 2 metastatic sites. hr[2]= Apatinib(850mg) vs. Placebo, hr[3]= Nivolumab vs.Placebo, hr[4]= Ramucirumab vs. Placebo, hr[5]= Trifluridine/tipiracil vs. Placebo, hr[6]= Apatinib(700mg) vs. Placebo, hr[7]= SLC vs. Placebo. SLC=salvage chemotherapy.

(D)Traces shown when hazard ratios (HRs) with 95% credible intervals (CIs) were used to assess OS for patients with more than 2 metastatic sites. hr[2]= Apatinib(850mg) vs. Placebo, hr[3]= Nivolumab vs.Placebo, hr[4]= Ramucirumab vs. Placebo, hr[5]= Trifluridine/tipiracil vs. Placebo, hr[6]= Apatinib(700mg) vs. Placebo, hr[7]= SLC vs. Placebo. SLC=salvage chemotherapy.

(E)Traces shown when hazard ratios (HRs) with 95% credible intervals (CIs) were used to assess OS for patients with no measurable disease. hr[2]= Nivolumab vs.Placebo, hr[3]= Ramucirumab vs. Placebo, hr[4]= Trifluridine/tipiracil vs. Placebo, hr[5]= Apatinib(700mg) vs. Placebo, hr[6]= SLC vs. Placebo; SLC=salvage chemotherapy.

(F)Traces shown when hazard ratios (HRs) with 95% credible intervals (CIs) were used to assess OS for patients with measurable disease. hr[2]= Nivolumab vs.Placebo, hr[3]= Ramucirumab vs. Placebo, hr[4]= Trifluridine/tipiracil vs. Placebo, hr[5]= Apatinib(700mg) vs. Placebo, hr[6]= SLC vs. Placebo; SLC=salvage chemotherapy.

**Fig. S4 Pooled hazard ratios for overall survival in subgroup patients** **without or with more than 2 metastatic sites.**


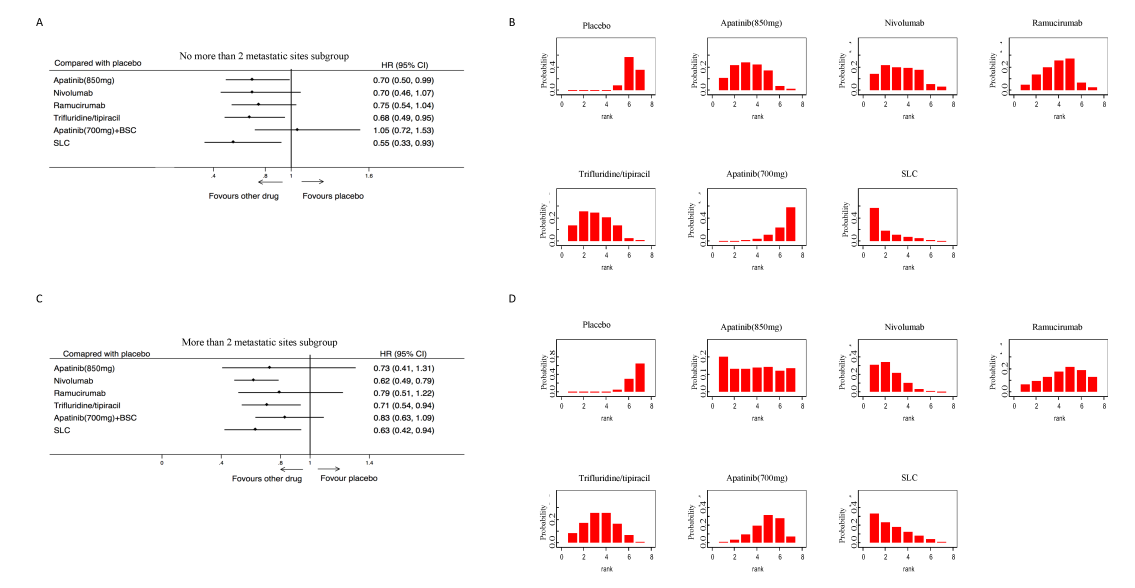


(A) Forest plot, with placebo as the comparator in patients with no more than 2 metastatic sites; A fixed effect model was adopted due to non-significant heterogeneity of publications (*I^2^* = 0.0%, p=0.562). (B) Ranking of treatments in terms of overall survival in patients with no more than 2 metastatic sites. (C) Forest plot, with placebo as the comparator in patients with more than 2 metastatic sites; A fixed effect model was adopted due to non-significant heterogeneity of publications (*I^2^* = 0.0%, p=0.722). (D) Ranking of treatments in terms of overall survival in patients with more than 2 metastatic sites. Rankograms were drawn according to distribution of the ranking probabilities. HR = hazard ratio. CI = credible interval. Numbers in parentheses indicate 95% credible intervals.

**Fig. S5 Pooled hazard ratios for overall survival in subgroup patients without or with measurable disease.**


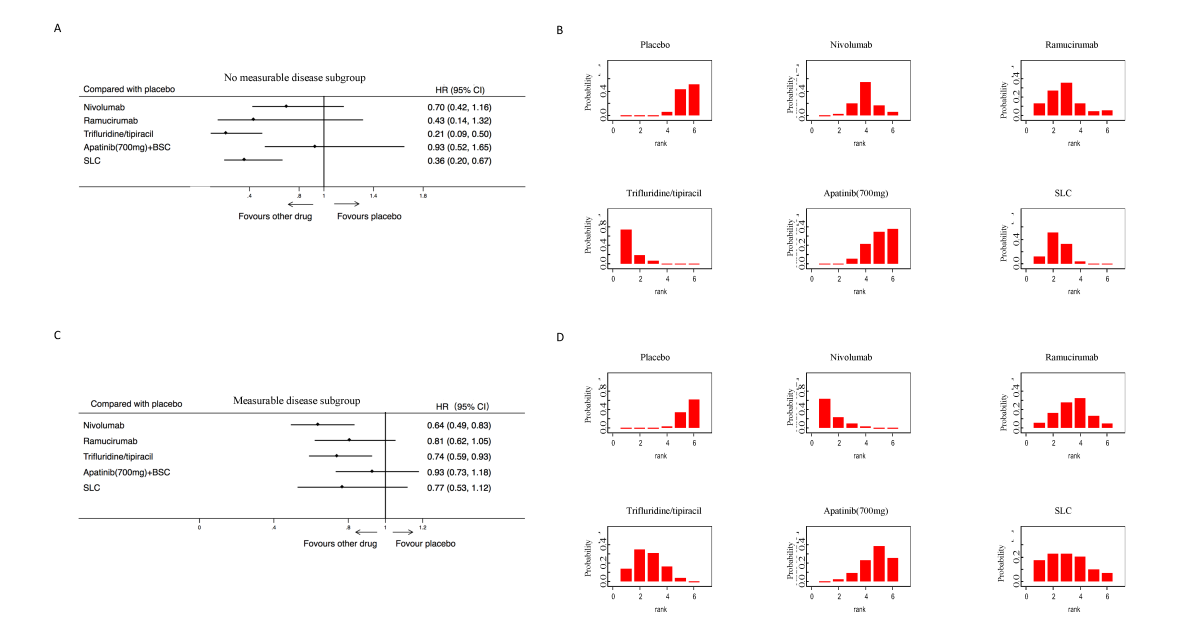


(A) Forest plot, with placebo as the comparator in patients with no measurable disease; significant heterogeneity of publications was seen (*I^2^* = 56.3%, p=0.057). (B) Ranking of treatments in terms of overall survival in patients with no measurable disease. (C) Forest plot, with placebo as the comparator in patients with more than measurable disease; A fixed effect model was adopted due to non-significant heterogeneity of publications (*I^2^* = 8.3%, p=0.359). (D) Ranking of treatments in terms of overall survival in patients with no measurable disease. Rankograms were drawn according to distribution of the ranking probabilities. HR = hazard ratio. CI = credible interval. Numbers in parentheses indicate 95% credible intervals.

**Fig. S6 Pooled hazard ratios for overall survival in subgroup patients with or without peritoneal metastasis.**


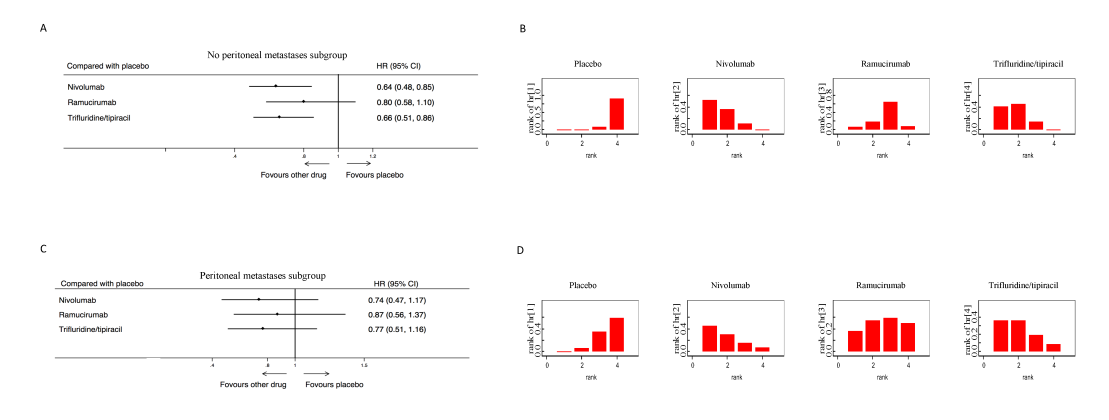


(A) Forest plot, with placebo as the comparator in patients with no peritoneal metastasis; A fixed effect model was adopted due to non-significant heterogeneity of publications (*I^2^* = 0.0%, p=0.586). (B) Ranking of treatments in terms of overall survival in patients with peritoneal metastasis. (C) Forest plot, with placebo as the comparator in patients with peritoneal metastasis; A fixed effect model was adopted due to non-significant heterogeneity of publications (*I^2^* = 0.0%, p=0.883). (D) Ranking of treatments in terms of overall survival in patients with peritoneal metastasis. Rankograms were drawn according to distribution of the ranking probabilities. HR = hazard ratio. CI = credible interval. Numbers in parentheses indicate 95% credible intervals.

**Fig. S7 Funnel plot of randomized controlled trials included in the meta-analysis for hazard ratios of overall survival(A) and Progression-free survival(B).**


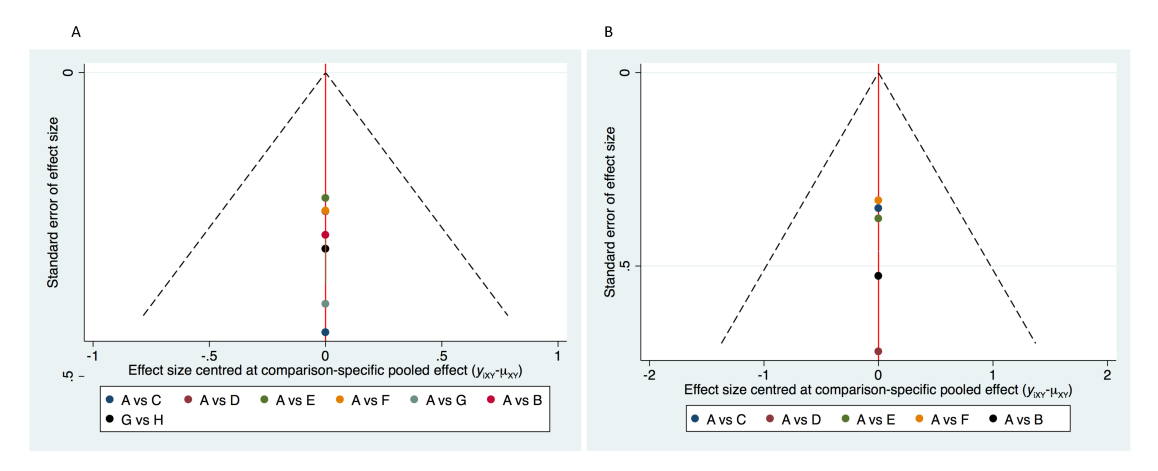


(A) A=placebo, B= Apatinib(850mg), C= Nivolumab, D= Ramucirumab, E= Trifluridine/tipiracil, F= Apatinib(700mg), G= SLC, H= Avelumab; SLC=salvage chemotherapy. (B) A=placebo, B= Apatinib(850mg), C= Nivolumab, D= Ramucirumab, E= Trifluridine/tipiracil, F= Apatinib(700mg).

**Fig. S8 Cochrane risk of bias tool assessment.**


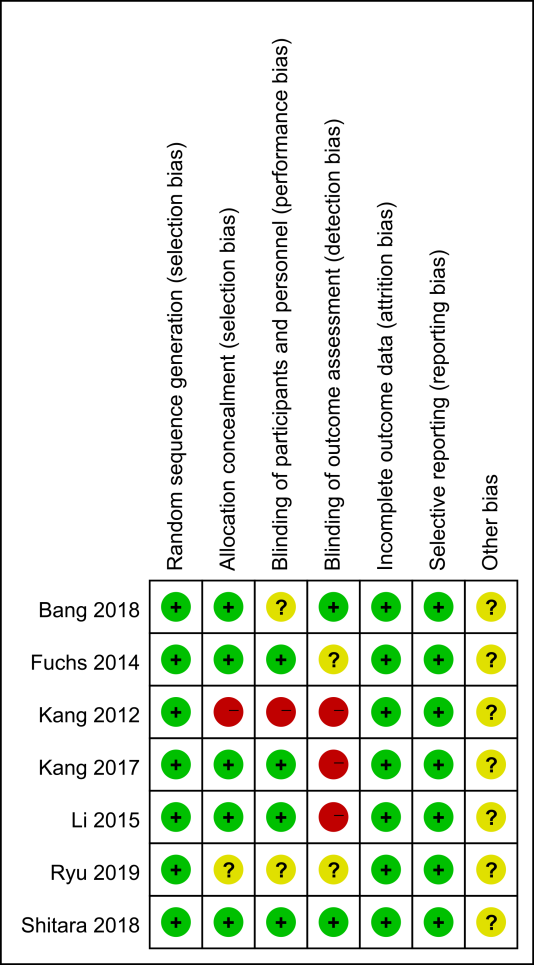

Supplement: Supplementary file 1 [file Table_1.DOCX]
